# Does Glaucoma Share Common Pathogenesis with Branch Retinal Vein Occlusion?

**DOI:** 10.1371/journal.pone.0156966

**Published:** 2016-06-15

**Authors:** Jong Chul Han, Doo Ri Eo, Taek Kwan Lee, Jong Hoon Shin, Changwon Kee

**Affiliations:** 1 Department of Ophthalmology, Samsung Medical Center, Sungkyunkwan University School of Medicine, Seoul, Republic of Korea; 2 Department of Ophthalmology, Busan National University Hospital, Busan National University School of Medicine, Busan, Republic of Korea; Bascom Palmer Eye Institute, University of Miami School of Medicine;, UNITED STATES

## Abstract

**Background:**

To evaluate the observed prevalence and the optic nerve head (ONH) characteristics of normal tension glaucoma (NTG)-suspect eyes in branch retinal vein occulusion (BRVO) eyes in Korean population.

**Methods:**

This was a retrospective observational study. We investigated 445 BRVO eyes that were diagnosed in the retina clinic of Samsung Medical Center between March 2005 and December 2011. The observed prevalence of NTG-suspect in BRVO eyes was evaluated compared to the previous population based study. In addition, NTG-suspect cases in BRVO were divided into three groups based on the characteristics of optic disc morphology.

**Results:**

In 445 BRVO eyes, 30 eyes were excluded from the present study. In 415 BRVO eyes, 4.3% (18 eyes) (95% confident interval [CI], 2.4–6.3%) were diagnosed with suspect glaucoma and this is not significantly different from the result in the general Korean population (*P* = 0.09). We classified the NTG-suspect eyes into three groups such as disc rim notching and thinning type (Group 1; 55.6%), optic cup-sited hemorrhage type (Group 2; 16.7%) and disc rim thinning and pallor type (Group 3; 27.8%). NTG-suspect in the fellow eye were only found in group 1 (80%) and group 2 (67%), but not in group 3 (*P* = 0.01).

**Conclusions:**

BRVO and glaucoma seem to have no common vascular pathogenesis in consideration of the prevalence of NTG-suspect in BRVO eyes compared to general Korean population.

## Introduction

Vascular factors that reduce ocular blood flow are independent risk factors for the development of glaucoma, especially normal tension glaucoma (NTG). Glaucoma might also be associated with vascular risk factors such as blood pressure [1,2], disc hemorrhage [3,4], vasospasm [1,5], and instability of ocular blood flow [6]. However, it has not been fully understood that incidence of NTG is related to vascular factors although such relationships have been suggested.

The correlations between retinal vein occlusion (RVO) and glaucoma has been reported previously [7–10]. The association between RVO and glaucoma has been explained by the cause-and-effect relationship associated with large cupping [9,11]. However, the possibility of common vascular pathogenesis between RVO and glaucoma has also been suggested [12–15]. Kim et al supported this hypothesis by proving RNFL loss in the fellow eye of patients with unilateral RVO [16]. The prevalence of glaucoma in RVO eyes may be higher than in the background population if the two diseases share a common vascular pathogenesis. CRVO eyes showed a higher glaucoma prevalence than uninvolved fellow eyes in a previous study [9]. Though topographical characteristics such as larger cup area or larger cup-to-disc ratio are known to be associated with BRVO eyes [11], the prevalence of glaucoma in BRVO eyes has not been studied yet. The diagnosis of glaucoma in BRVO eyes is not practical because RNFL thickness may be decreased in sectors with RVO [16], thus this study sought to examine the prevalence and characteristics of glaucoma suspect eyes with the intraocular pressure (IOP) less than 21 mmHg (NTG-suspect eyes) in the Korean BRVO.

## Materials and Methods

This study was performed at a single center according to the tenets of the Declaration of Helsinki. The study was approved by the institutional review board of the Samsung Medical Center.

Chart reviews of 445 subjects diagnosed with BRVO in the retina clinic of Samsung Medical Center between March 2005 and December 2011 were performed. Patient characteristics, such as age, gender and history of hypertension, diabetes mellitus (DM) and hypercholesterolemia were recorded at the first visit. Slit-lamp examination and IOP by Goldmann tonometer results were recorded. Mean IOP and peak IOP during the follow-up period were calculated. Mean IOP was defined as the average IOP values during the observational periods. Peak IOP was defined as the highest value among the recorded IOPs. Initial fundus photos and evaluation of optic nerve head (ONH) such as the vertical cup-to-disc ratio were used for the diagnosis of BRVO and glaucoma suspect. The vertical cup-to-disc ratio was measured using Image J (version 1.52; National Institutes of Health, Bethesda, MD, USA).

BRVO was diagnosed using following criteria: retinal vein occlusion at an arteriovenous crossing outside the optic disc or at an optic cup, upstream venous congestion, intraretinal hemorrhage, edema, cotton wool spots, and vein-to-vein collaterals with adjacent branch veins in the absence of similar changes in the surrounding venous drainage units [17]. Two venous obstruction sites—the arteriovenous (AV) crossing site and the optic cup site—were defined in this study [10]. The occlusion sites were identified by an abrupt change in the caliber of the obstructed vein because of dilatation distal to the occlusion and proximal attenuation.

NTG-suspect eyes were diagnosed by two glaucoma specialist (JCH and JHS). In the present study, the visual field data were not unavailable because the BRVO eyes were enrolled from retina clinic. In addition, only NTG eyes were included, thus we defined NTG-suspect eyes using the new diagnostic criteria (optic disc-NTG suspect criteria). Based on the International Society for Geographical and Epidemiological Ophthalmology (ISGEO) criteria [18], the new diagnostic criteria of NTG-suspect eyes with BRVO were defined. Most of the BRVO eyes had superotemporal and inferotemporal RNFL defect associated with venous obstruction, thus the presence of a RNFL defect was eliminated from the new diagnostic criteria. The new diagnostic criteria for NTG-suspect eyes in BRVO included: a vertical cup-to-disc ratio of the ONH of ≥ 0.7; rim width at the superior (11–1 hours) or inferior portion (5–7 hours) 0.05–0.1 times the disc diameter, and a difference in the vertical cup-to-disc ratio of 0.3 between both eyes. In addition, the optic disc rim pallor with thinning was also considered consistent with glaucoma suspect. Exclusion criteria included the following: (1) a history of IOP elevation ≥ 21 mmHg, (2) a history of ocular surgery except for uncomplicated cataract surgery, or (3) a history of ocular disease, such as uveitis, complicated DM retinopathy, or neovascular glaucoma (S1 Table).

NTG-suspect eyes were classified based on the clinical characteristics into three groups. Group 1 included the eyes with dominant rim notching or thinning. Group 2 included the eyes with optic cup-sited BRVO and group 3 included eyes that have rim pallors without rim notching or thinning. In cases where the eyes were difficult to classify, the decision was made after a consensus was reached by 2 investigators (JCH and CK). To understand the characteristics of each group, age, mean IOP, cup-to-disc ratio, rim morphology (notching, thinning and pallor) and presence of glaucoma-suspect in the fellow eyes among the groups.

### Statistical Analysis

Statistical analyses were performed using a commercially available software package (SPSS ver. 18.0 for Windows; SPSS Inc., Chicago, IL, USA). An independent t-test or Mann-Whitney *U* test was performed to compare baseline characteristics between patients with BRVO with and without NTG-suspect. The Kruskal-Wallis test was performed to compare among the three subgroups. Categorical variables were compared using the chi-square test or Fisher’s exact test. The exact binomial test was used to compare the prevalence of NTG-suspect eyes in patients with BRVO and the general population based on data from a previously published study. A *P* ≤ 0.05 was considered statistically significant.

## Results

Of the 445 BRVO patients, 30 eyes from 30 patients were excluded due to accompanying ocular history such as DM retinopathy or uveitis, and 415 eyes from 415 patients were included in the study. NTG-suspect eyes were present in approximately 4.3% of Korean BRVO eyes. The mean age was 62.1, 65.7 and 62.0 years and the proportion of male patients was 53.3%, 33.3% and 54.2% across all BRVO patients, and in the group of BRVO patients with and without NTG-suspect eyes, respectively (*P* = 0.34; *P* = 0.13). The hypertension rate was 40.2%, 44.4% and 38.4%, the DM rate was 30.4%, 22.2% and 29.5%, and the hypercholesterolemia rate was 31.8%, 38.9%, and 31.5% across all BRVO patients, and in the group of BRVO patients with and without NTG-suspect eyes, respectively (*P* = 0.57; *P* = 0.53; *P* = 0.60) (Table 1).

**Table 1:** Demographic and systemic characteristics of included branch retinal vein occlusion with and without glaucoma suspect.

	Total BRVO patients	with glaucoma suspect	without glaucoma suspect	*P*-value
Patient, n (%)	415 (100%)	18 (4.3%)	397 (95.7%)	
Age (year)	62.1± 7.5	65.7± 9.6	62.0 ± 7.5	0.34*
Male gender	221 (53.3%)	6 (33.3%)	215 (54.2%)	0.13^†^
Systemic factor				
HTN, n (%)	167 (40.2%)	8 (44.4%)	159 (38.4%)	0.57^†^
DM, n (%)	126 (30.4%)	4 (22.2%)	122 (29.5%)	0.53^†^
Hypercholesterolemia, n (%)	132 (31.8%)	7 (38.9%)	125 (31.5%)	0.60^†^

BRVO = branch retinal vein occlusion; IOP = intraocular pressure; HTN = hypertension; DM = diabetes mellitus.

*Comparison using independent *t*-test between BRVO with and without glaucoma suspect.

^†^Comparison using chi-square test or Fisher’s exact test between BRVO with and without glaucoma suspect.

The mean IOP and peak IOP was 15.6, 16.1 and 15.5 mmHg and 16.5, 16.5 and 16.3 mmHg, respectively (*P* = 0.25; *P* = 0.64). The vertical cup-to-disc ratio was significantly larger in patients with BRVO with NTG-suspect eyes than in those without NTG-suspect eyes (0.8 ± 0.1 vs 0.5 ± 0.1, *P* = 0.003). The optic cup-type venous obstruction was found in 17 BRVO patients (4.1%) and there was a significant difference in the presence of optic cup-type venous obstruction between glaucoma-suspect and non-NTG-suspect eyes in patients with BRVO (*P* = 0.03). For arteriovenous crossing-type venous obstructions, the superotemporal venous obstruction type was more common than the inferotemporal type in patients with BRVO with and without NTG-suspect eyes, and there were no significant differences (Table 2).

**Table 2:** Ocular parameters and the characteristics of venous obstructions in branch retinal vein occlusion with and without glaucoma suspect.

	Total BRVO patients (N = 415)	with glaucoma suspect (N = 18)	without glaucoma suspect (N = 397)	*P*-value*
Ocular factor				
Mean IOP (mmHg)	15.6 ± 2.7	16.1 ± 2.1	15.5 ± 2.7	0.25*
Peak IOP (mmHg)	16.5 ± 3.0	16.5 ± 2.7	16.3 ± 3.0	0.64*
Vertical cup-to disc ratio	0.5 ± 0.1	0.8 ± 0.1	0.5 ± 0.1	0.003*
Venous obstruction type				
Optic cup type, n (%)	17 (4.1%)	3 (16.7%)	14 (3.5%)	0.03^†^
AV crossing type, n (%)	398 (95.9%)	15 (83.3%)	383 (96.5%)	
Superotemporal, n (%)	235 (56.6%)	9 (50.0%)	226 (56.9%)	1.00^‡^
Inferotemporal, n (%)	163 (39.3%)	6 (33.3%)	157 (39.5%)	

BRVO = branch retinal vein occlusion; IOP = intraocular pressure; HTN = hypertension; DM = diabetes mellitus.

*Comparison using independent t-test or Mann-Whitney U test between BRVO with and without glaucoma suspect.

^†^Comparison of venous obstruction type using Fisher’s exact test between BRVO with and without glaucoma suspect.

^‡^Comparison of the location of arteriovenous obstruction using chi-square test between BRVO with and without glaucoma suspect.

The age-specific prevalence rates of glaucoma-suspect are summarized in Table 3. The overall prevalence of NTG-suspect eyes in BRVO in the present study was 4.3% (95% CI, 2.4%–6.3%). The prevalence of NTG-suspect eyes in BRVO was less than that of POAG including suspected cases ≤ 21 mmHg reported by Kim et al in the Korean general population, however, this was not statistically significant (*P* = 0.09) (Table 3).

**Table 3:** Comparison of the prevalence of glaucoma suspects in branch retinal vein occlusion with the prevalence reported in the published study in the Korean population (Namil study).

Age Group (year)	In Branch Retinal Vein Occlusion	Population Based Study	*P*-value*
	Observed Prevalence of Glaucoma Suspect	Prevalence of POAG Including Glaucoma Suspect (Irrespective of IOP)	
40–49	3.7% (0.0%–9.3%)	3.8% (1.2%–6.4%)	0.37
50–59	4.9% (0.0%–12.2%)	3.4% (1.4%–5.4%)	0.25
60–69	4.5% (1.9%–8.3%)	6.3% (4.0%–8.6%)	0.09
70–79	4.3% (1.4%–7.9%)	6.7% (4.4%–9.0%)	0.07
≥80	3.3% (0.0%–13.0%)	8.9% (3.8%–14.0%)	0.18
Total	4.3% (2.4%–6.3%)	5.7% (4.5%–6.9%)	0.04
	Observed Prevalence of Glaucoma Suspect	Prevalence of POAG Including Suspected Cases (with ≤ 21mmHg)	
Total	4.3% (2.4%–6.3%)	4.8% (3.9%–5.7%)	0.09

IOP = intraocular pressure; POAG = primary open-angle glaucoma.

* *P*-values were calculated using the exact binomial test.

**Fig 1:**
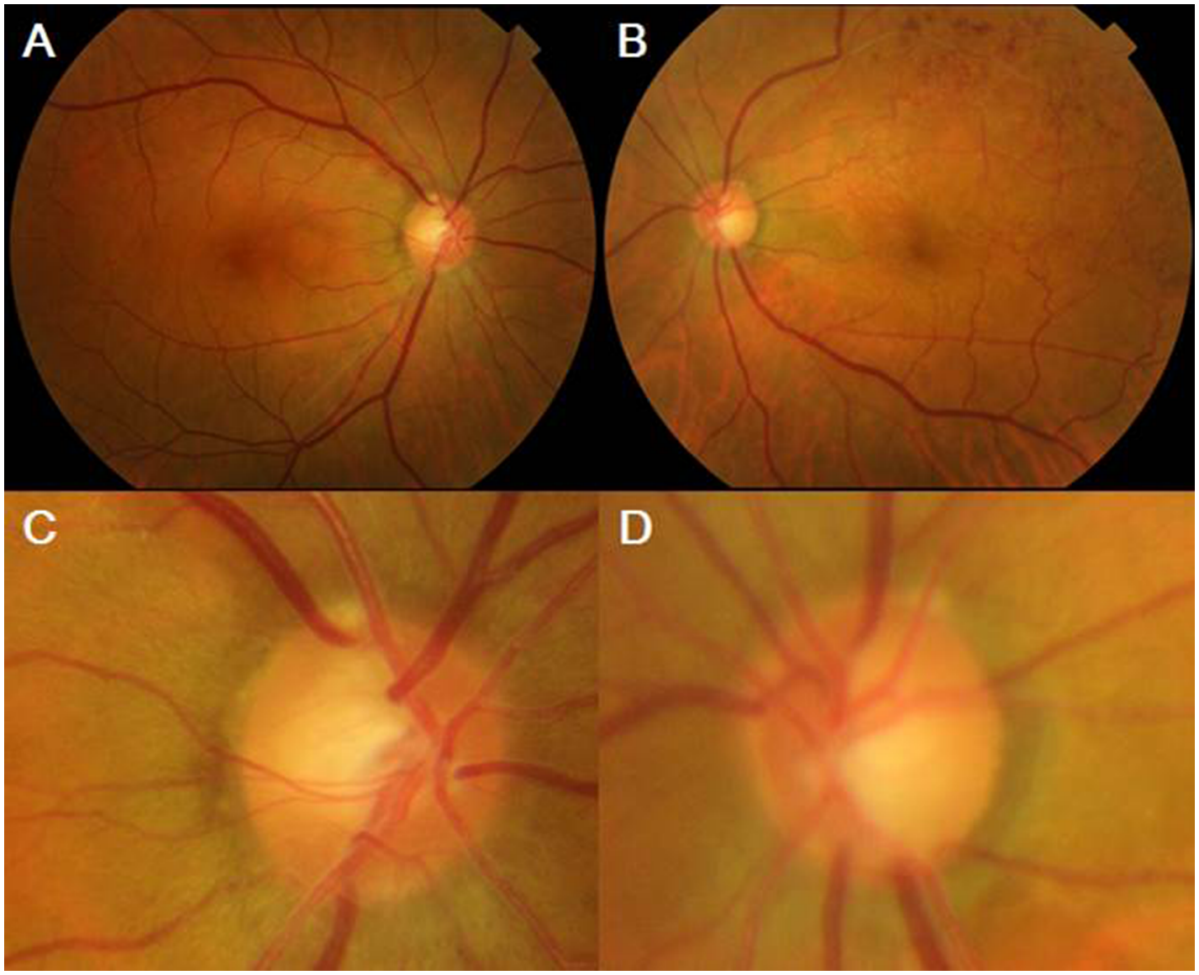
A case of rim notching type (Group 1). (A, C) A fundus photo of the right eye shows the retinal nerve fiber layer (RNFL) defect and rim notching at the superotemporal area. (B,D) A fundus photo of the left eye shows the superotemporal retinal hemorrhage, RNFL defect and rim notching at the superotemporal and inferotemporal areas.

**Fig 2:**
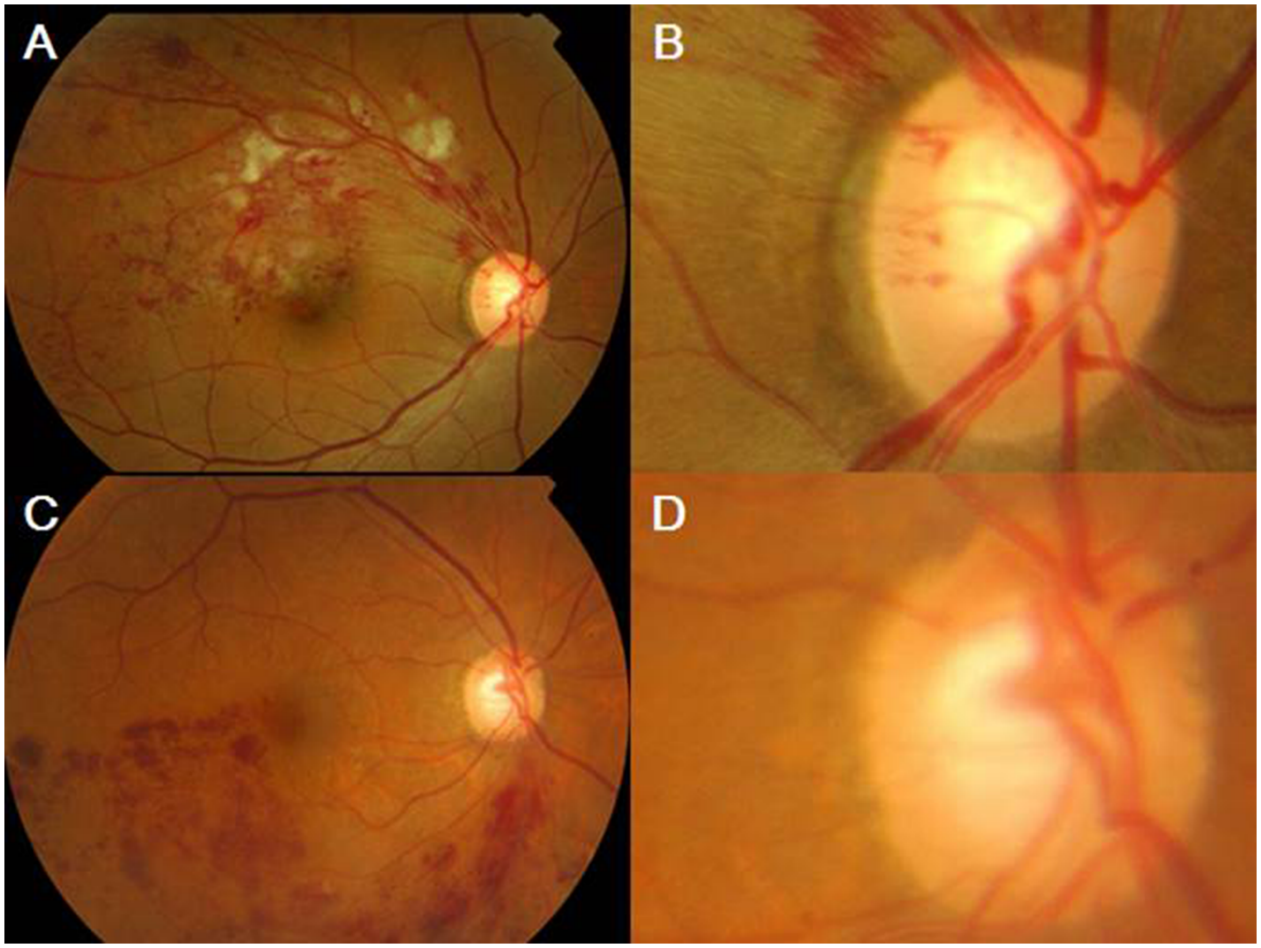
A case of optic cup-sited BRVO type (Group 2). (A, B) A fundus photo of the right eye shows the optic cup-sited branch retinal vein occlusion (BRVO) at superotemporal area. The rim notching has the same orientation at the the retinal hemorrhage. (C,D) A fundus photo of the right eye shows the optic cup-sited BRVO at the inferotemporal area. The rim notching has the same orientation as the retinal hemorrhage.

**Fig 3:**
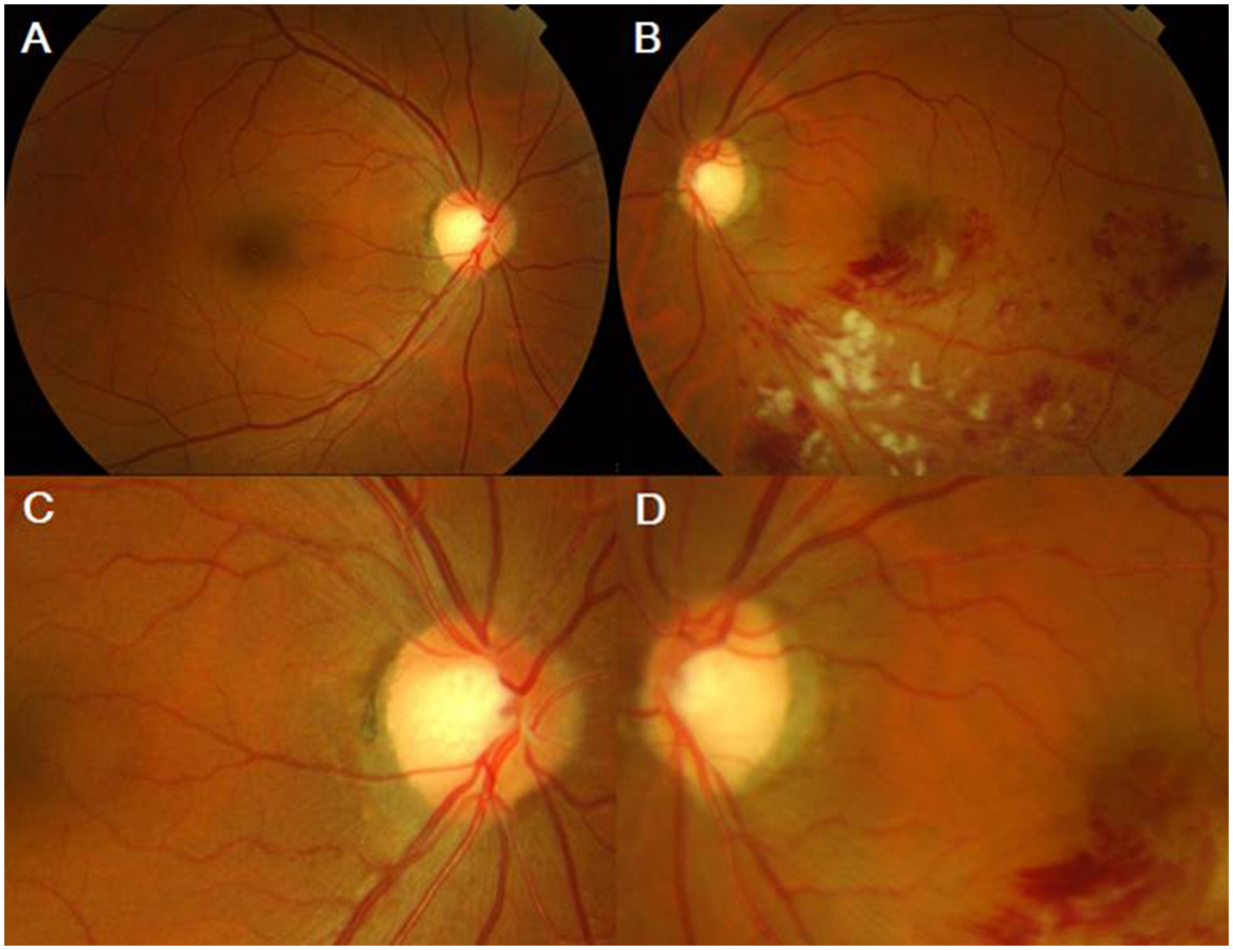
A case of rim pallor type (Group 3). (A, C) A fundus photo of the right eye shows normal findings. (B,D) A fundus photo of the left eye reveals inferotemporal BRVO at the arteriovenous crossing site. The optic disc shows thinning and rim pallor with the same direction.

When the NTG-suspect eyes were classified into sub-groups, 10 cases, 3 cases and 5 cases were included in group 1 (rim notching/thinning type), group 2 (optic cup-sited BRVO type) and group 3 (rim pallor type). Among the groups, no significant differences were found in age, mean IOP, the cup-to-disc ratio (*P* = 0.79; *P* = 0.17; *P* = 0.13). In group 1 (rim notching/thinning type), glaucoma-suspect fellow eye was found in 80% of cases (Fig 1). Group 2 (optic cup-sited BRVO type) showed similar clinical characteristics with group 1(Fig 2). Three cases in group 2 showed the corresponding venous obstruction locations compared to the rim notching sites. All the cases in group 3 showed rim pallor instead of rim notching or thinning, and showed no glaucoma-suspect cases in the fellow eyes (Fig 3; Table 4).

**Table 4:** The different clinical characteristics among the groups.

	Group 1 (Rim notching/thinning)	Group 2 (Optic cup-sited BRVO)	Group 3 (Rim pallor)	*P*-value
Case (N)	10 (55.6%)	3 (16.7%)	5 (27.8%)	
Age (years), range	65 (49–77)	69 (52–82)	65 (61–72)	0.79*
Mean IOP (mmHg), range	18 (13–20)	17 (16–18)	15 (10–17)	0.17*
Cup-to-disc ratio, range	0.8 (0.5–0.9)	0.7 (0.6–0.8)	0.7 (0.5–0.8)	0.13*
Rim notching	7 (70%)	2 (67%)	0 (0%)	0.03^†^
Rim thinning	3 (30%)	1 (33%)	0 (0%)	0.43^†^
Rim pallor	2 (20%)	1 (33%)	5 (100%)	0.01^†^
Glaucoma suspect in opposite eye	8 (80%)	2 (67%)	0 (0%)	0.01^†^

IOP = intraocular pressure.

*Comparison using Kruskal-Wallis test among the subgroups.

^†^Comparison using chi-square test or Fisher’s exact test among the subgroups.

## Discussion

This is the first study to investigate the prevalence of NTG-suspect eyes in Korean BRVO eyes. The observed prevalence of NTG-suspect eyes in BRVO eyes was approximately 4.3%. Because we included the glaucoma-suspect eyes ≤ 21 mmHg (NTG-suspect eyes) in the present study, the prevalence of the present study was compared to that of the OAG with IOP ≤ 21 mmHg in the previous study. In Korean population, the prevalence of open angle glaucoma suspects with ≤ 21 mmHg was 4.8%. In the present study, the prevalence of NTG-suspect eyes in patients with BRVO did not showed significant difference comparing to that in general Korean population.

In clinic, disc pallor associated with ischemic optic neuropathy occasionally seems like glaucomatous disc [19–21]. It would increase the sensitivity of glaucoma-suspects if all disc pallor cases are regarded as the glaucoma-suspects. However, even after including optic disc pallor cases in glaucoma-suspect diagnosis, the prevalence of NTG-suspect in BRVO eyes was not higher than that of the general Korean population. If BRVO and glaucoma share common vascular pathogenesis, the glaucoma-suspect cases in BRVO eyes would predominate over normal populations. Therefore, it is speculated that the comorbidity of the two diseases may not be strongly related to a common vascular pathogenesis. This is in contrast to a previous study suggesting a common vascular pathogenesis between glaucoma and RVO. Kim et al showed RNFL thinning at superotemporal and inferotemporal sector in the fellow eye of RVO patients. Because the fellow eyes of RVO patients had RNFL thinning at the similar location to RVO eyes, it was thought that there might be a common mechanism between RVO and glaucoma [16]. However, considering that RNFL loss can develop in response to systemic risk factors such as DM and systemic hypertension [22], the result of the previous study could be from the systemic risk factors rather than glaucoma. To understand whether the RNFL thinning of the fellow eye in BRVO is a true glaucomatous change, further prospective studies are warranted.

Although the present study showed no significant difference in the prevalence of NTG-suspect eyes in BRVO patients compared to the known prevalence in the general population, BRVO patients with NTG-suspect eyes were subclassified into several groups to determine whether unique characteristics could be identified. The majority of the rim notching-type cases (group 1) are likely to be glaucoma based on the observation of glaucomatous defects in the fellow eyes in 80% of the group 1. Glaucoma eyes have higher chance to be diagnosed with glaucoma in the opposite eyes [23]. However, given that the prevalence of NTG-suspect eyes in patients with BRVO was not significantly different from that of the general population, glaucoma-suspect features are likely to have been present regardless of the presence of BRVO in group 1.

Previously, glaucoma has been noted to be a risk factor in the development of RVO associated with large cup-to-disc ratio and high IOP. In the Beaver Dam Eye Study, glaucoma and a high cup-to-disc ratio were found significantly more frequently in RVO eyes than in the normal population [24]. In the Singapore Indian Eye Study, the BRVO prevalence was higher in the larger cup-to-disc ratio irrespective of the presence of glaucoma [11]. This could be due to the fact that retinal vein is distorted as it bends at the sharpened rim of the large optic disc cup as suggested previously [10]. Kim et al showed that an optic cup-sited RVO was more strongly associated with glaucomatous changes in the fellow eye than arteriovenous crossings-sited RVO [25]. Beaumont et al also found that an optic cup-sited RVO was associated with a larger cup-to-disc ratio and higher IOP than an arteriovenous crossing-sited RVO [10]. In the present study, there were optic cup-sited obstructions in 16.7% of BRVO patients with NTG-suspect eyes, but in 3.5% of BRVO patients without NTG-suspect eyes. In addition, all the three optic cup-sited RVO cases were found to have retinal hemorrhage sites corresponding to the locations of optic disc notching (group 2). Therefore, it can be postulated that optic cup-sited venous obstruction may be more associated with large disc cupping with retinal vein distortion than arteriovenous crossing-sited venous obstruction in BRVO eyes. However, it seems that optic cup-sited BRVOs are not common, it did not increase the prevalence of NTG-suspect eyes in BRVO. It is not clear why optic cup-sited BRVO were not found more commonly in NTG-suspect eyes. One possible hypothesis is that many the optic cup-sited RVOs might appear as central- or hemi-RVO rather than BRVOs, and thus might have not been included in the present study.

Diagnosing glaucoma in RVO is not easy because RVO can affect independently affect RNFL thickness [26]. In addition, RVOs sometimes induce a more pallorous optic disc margin and mimic glaucoma. RVO can also cause ischemic changes in the optic nerve head. In a previous study, optic disc pallor in CRVO presented with an ischemic patterm in 38% of cases compared to 2% of cases with a non-ischemic type at 1 year after venous obstruction [27]. The previous BRVO study showed optic disc pallor in approximately 14% of cases at 1 year and 21% of cases at 3 years from the onset of BRVO [27]. In the present study, definite disc pallor was present in about 2% of the total BRVO cases. This difference among the studies may be due to the subjective nature of the disc evaluation and differences in the study population. To increase detection sensitivity, BRVO eyes with rim pallor were included as NTG-suspect cases. However, group 3 (optic disc with rim pallor) had no glaucoma-suspect cases in fellow eyes, whereas group 1 and group 2 showed a prevalence of 89% and 67% of glaucoma-suspect fellow eyes. This means that Group 3 may not have true glaucomatous changes, but have glaucomatous-like changes at ONH due to ischemic damages of venous occlusion.

The present study has some limitations. First, this study was designed as a retrospective chart review of BRVO patients. In some cases, the temporal relations between NTG-suspect and BRVO were not clear, and this could limit inferences regarding the causal relationship between the two diseases. Second, BRVO patients from a single hospital were included, which may have introduced selection bias. However, based on the premise that hospital based studies usually show a larger disease prevalence than general population studies, the prevalence of the present study still seems to be small. Third, only fundus photos were used to evaluate the optic disc in this study. Another diagnostic approach such as optical coherence tomography or Heidelberg retinal topography could help us explore the characteristics of ONH in NTG-suspect eyes in patients with BRVO. Fourth, IOP was not corrected by central corneal thickness (CCT) in the present study. Because the present study investigated the prevalence of NTG-suspect eyes based on the peak IOP, exact IOP is important. It could influence on the values of IOPs though it is not certain how much it affected.

In conclusion, BRVO and glaucoma are unlikely to have a strong common vascular pathogenesis because the observed prevalence of NTG-suspect eyes in patients with BRVO was similar to that of the general Korean population. In addition, NTG-suspect eyes could be divided into three groups based on clinical characteristics and this classification might be helpful in understanding the clinical association between BRVO and glaucoma.

## Supporting Information

S1 Fig The distributions of continuous parameters of total branch retinal vein occlusion patients. (PNG)

S1 Table Comparisons between the International Society of Geographical and Epidemiological Ophthalmology (ISGEO) criteria and optic disc-normal tension glaucoma (NTG) suspect criteria. (DOCX)

S2 Table The age distributions of the branch retinal vein occlusion (BRVO) patients. (DOCX)

S3 Table Demographics of the normal-tension glaucoma suspects with branch retinal vein occlusion. (DOCX)
