# Supplementary figures and images for: Does Glaucoma Share Common Pathogenesis with Branch Retinal Vein Occlusion?
Source: PLoS One. 2016 Jun 15;11(6):e0156966. doi: 10.1371/journal.pone.0156966 (PMC4909192; doi:10.1371/journal.pone.0156966)

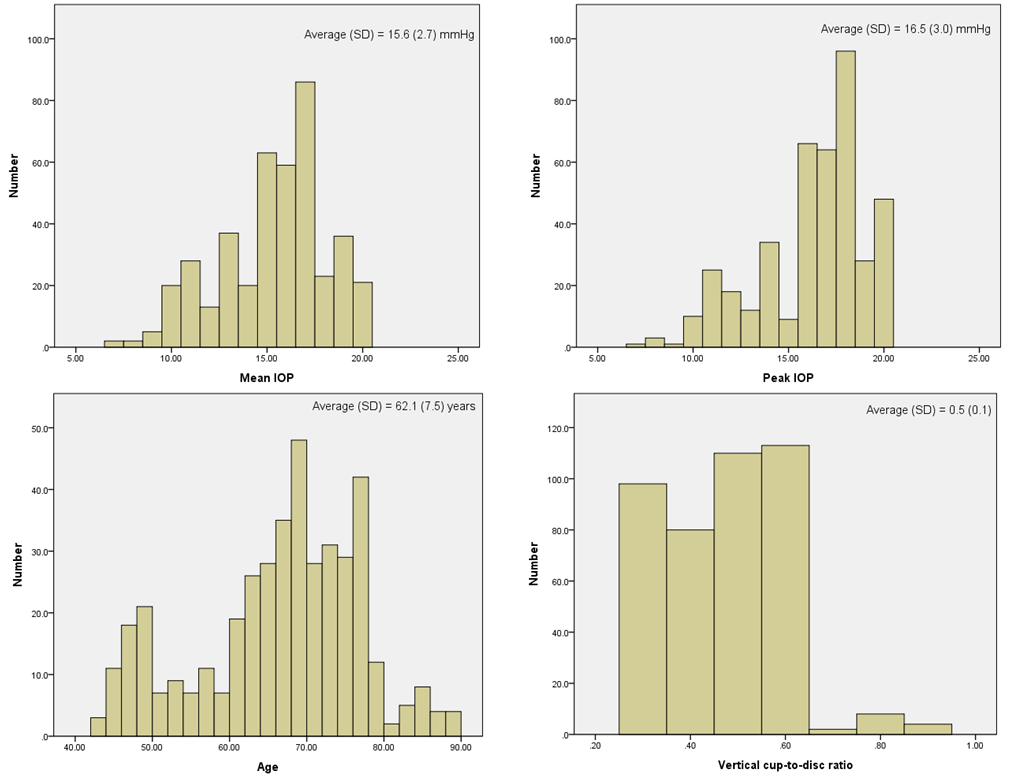

Supplement: S1 Fig — (PNG) [file pone.0156966.s001.png]
