# Supplementary material for: Does Glaucoma Share Common Pathogenesis with Branch Retinal Vein Occlusion?
Source: PLoS One. 2016 Jun 15;11(6):e0156966. doi: 10.1371/journal.pone.0156966 (PMC4909192; doi:10.1371/journal.pone.0156966)
Supplement: S1 Table — (DOCX) [file pone.0156966.s002.docx]

**S1 Table. Comparisons between the International Society of Geographical and Epidemiological Ophthalmology (ISGEO) criteria and optic disc-normal tension glaucoma (NTG) suspect criteria**

| ISGEO criteria (for glaucoma) | **Category 1 diagnosis** (structural and functional evidence)  Eyes with a CDR or CDR asymmetry ≥97.5th percentile for the normal population, or a neuroretinal rim width reduced to ≤0.1 CDR (between 11 to 1 o'clock or 5 to 7 o'clock) that also showed a definite visual field defect consistent with glaucoma.  **Category 2 diagnosis** (advanced structural damage with unproved field loss)  If the subject could not satisfactorily complete visual field testing but had a CDR or CDR asymmetry ≥ 99.5th percentile for the normal population, glaucoma was diagnosed solely on the structural evidence.  In diagnosing category 1 or 2 glaucoma, there should be no alternative explanation for CDR findings (dysplastic disc or marked anisometropia) or the visual field defect (retinal vascular disease, macular degeneration, or cerebrovascular disease).  **Category 3 diagnosis** (Optic disc not seen. Field test impossible)  If it is not possible to examine the optic disc, glaucoma is diagnosed if: (A) The visual acuity <3/60 and the IOP >99.5th percentile, or (B) The visual acuity <3/60 and the eye shows evidence of glaucoma filtering surgery, or medical records were available confirming glaucomatous visual morbidity. |
| --- | --- |
| Optic disc-NTG suspect criteria | **Diagnosis of glaucoma suspect (structural damage without proved field loss)**  A vertical cup-to-disc ratio of the optic nerve head of ≥ 0.7 or  Rim width at the superior (11-1 hours) or inferior portion (5-7 hours) 0.05-0.1 times the disc diameter or  A difference in the vertical cup-to-disc ratio of 0.3 between both eyes or  Optic disc rim pallor with thinning  No history of IOP elevation ≥ 21 mmHg and  No history of ocular surgery except for uncomplicated cataract surgery and  No history of ocular disease such as uveitis or complicated diabetes mellitus (DM) retinopathy or neovascular glaucoma |
