# Supplementary material for: Does Glaucoma Share Common Pathogenesis with Branch Retinal Vein Occlusion?
Source: PLoS One. 2016 Jun 15;11(6):e0156966. doi: 10.1371/journal.pone.0156966 (PMC4909192; doi:10.1371/journal.pone.0156966)
Supplement: S2 Table — (DOCX) [file pone.0156966.s003.docx]

| Age (years) | Total BRVO patients | with glaucoma suspect | without glaucoma suspect |
| --- | --- | --- | --- |
| 40 – 49 | 54 | 2 | 52 |
| 50 – 50 | 41 | 2 | 39 |
| 60 – 69 | 157 | 7 | 150 |
| 70 – 79 | 140 | 6 | 134 |
| 80 - | 23 | 1 | 22 |
|  | 415 | 18 | 397 |

**S2 Table. The age distributions of the branch retinal vein occlusion (BRVO) patients**
