# Supplementary material for: Does Glaucoma Share Common Pathogenesis with Branch Retinal Vein Occlusion?
Source: PLoS One. 2016 Jun 15;11(6):e0156966. doi: 10.1371/journal.pone.0156966 (PMC4909192; doi:10.1371/journal.pone.0156966)
Supplement: S3 Table — (DOCX) [file pone.0156966.s004.docx]

|  | **Group** | **Laterality** | **Age (years)** | **Sex** | **DM** | **HTN** | **Peak IOP (mmHg)** | **Cup-to-disc ratio** | **Rim defect** | **Rim pallor** | **RNFL defect in the fellow eye** | **Obstruction site** |
| --- | --- | --- | --- | --- | --- | --- | --- | --- | --- | --- | --- | --- |
| 1 | 1 | R | 62 | M | - | + | 20 | 0.8 | notching | - | + | AV |
| 2 | 1 | L | 49 | M | - | - | 20 | 0.8 | notching | - | + | AV |
| 3 | 3 | L | 63 | F | + | - | 16 | 0.8 | - | + | - | AV |
| 4 | 1 | L | 77 | F | - | + | 17 | 0.9 | notching | - | + | AV |
| 5 | 1 | L | 58 | F | - | + | 16 | 0.8 | notching | - | + | AV |
| 6 | 3 | L | 69 | F | + | - | 17 | 0.8 | - | + | - | AV |
| 7 | 1 | L | 76 | F | - | + | 18 | 0.9 | notching | + | - | AV |
| 8 | 3 | L | 72 | M | - | + | 10 | 0.7 | - | + | - | AV |
| 9 | 1 | L | 69 | M | + | + | 13 | 0.9 | thinning | - | + | AV |
| 10 | 2 | R | 52 | F | - | + | 16 | 0.6 | notching | + | + | Disc |
| 11 | 1 | L | 70 | F | + | + | 20 | 0.9 | thinning | - | + | AV |
| 12 | 3 | R | 61 | F | - | + | 17 | 0.6 | - | + | - | AV |
| 13 | 3 | L | 61 | M | + | - | 13 | 0.6 | - | + | - | AV |
| 14 | 1 | L | 65 | F | + | + | 20 | 0.8 | notching | - | - | AV |
| 15 | 2 | L | 82 | F | - | + | 16 | 0.7 | thinning | - | + | Disc |
| 16 | 1 | R | 49 | M | - | + | 13 | 0.5 | thinning | + | + | AV |
| 17 | 1 | R | 73 | F | - | + | 18 | 0.8 | notching | - | + | AV |
| 18 | 2 | R | 74 | F | - | + | 18 | 0.8 | notching | - | - | Disc |

**S3 Table. Demographics of the normal-tension glaucoma suspects with branch retinal vein occlusion**
